# C-Type Lectin Receptor Dectin-2 Binds to an Endogenous Protein β-Glucuronidase on Dendritic Cells

**DOI:** 10.1371/journal.pone.0169562

**Published:** 2017-01-03

**Authors:** Daiki Mori, Kensuke Shibata, Sho Yamasaki

**Affiliations:** 1 Division of Molecular Immunology, Medical Institute of Bioregulation, Kyushu University, Fukuoka, Japan; 2 Department of Molecular Immunology, Medical Mycology Research Center, Chiba University, Chiba, Japan; National Institute of Infectious Diseases, JAPAN

## Abstract

C-type lectin receptors (CLRs) recognize pathogen-derived ligands and abnormal self that trigger protective immune responses. However, the precise nature of self ligands recognized by CLRs remains to be determined. Here, we found that Dectin-2 recognizes bone marrow-derived dendritic cells (BMDCs) using Dectin-2-expressing reporter cells. This activity was inhibited by an excessive amount of mannose, and by the mutation of mannose-binding motif in Dectin-2. β-glucuronidase (Gusb) was identified as a protein bound to Dectin-2 and mutations of N-glycosylation sites in Gusb impaired the binding of Gusb to Dectin-2. Overexpression of Gusb in a macrophage cell line conferred an ability to stimulate Dectin-2-expressing reporter cells. Our study suggests that a glycosylated protein with mannose-related structure is recognized by Dectin-2.

## Introduction

Innate immune responses are initiated by pattern recognition receptors (PRRs) such as Toll-like receptors (TLRs), NOD-like receptors, RIG-I like helicase and C-type lectin receptors (CLRs) that recognize pathogen-associated molecular patterns (PAMPs) [1]. C-type lectins are defined as a family of proteins that bind carbohydrates in the presence of calcium. CLRs comprise at least one carbohydrate recognition domain (CRD) that in most cases binds sugars [2]. After recognition of ligands, CLRs activate innate immune responses via an integral immunoreceptor tyrosine-based activation motif (ITAM) in the cytoplasmic tail or via association with ITAM-bearing adaptor molecules such as FcRγ and DAP-12 [3].

Historically, many studies have been focused on identification of non-self ligands because the first ITAM-coupled CLR ligand was identified in pathogens [4]. Following this finding, β-glucan, α-mannan and cord factor trehalose 6,6'-dimycolate have been identified as ligands for Dectin (DC-associated C-type lectin)-1, Dectin-2 and Macrophage inducible C-type lectin (Mincle) respectively [4–10]. More recently, we identified mannose-capped lipoarabinamannan (Man-LAM) on mycobacteria as a ligand for Dectin-2 [11]. These CLRs consist of a cytoplasmic domain, a putative transmembrane domain, a stalk domain and a CRD, which is mainly involved in ligand recognition. In contrast to the pathogen-derived ligands, only a few endogenous molecules are shown to be recognized by CLRs including C-type lectin domain family 9 member A (Clec9a) and Mincle [12–15] and endogenous ligands for Dectin-2 have not been identified so far.

It is widely accepted that glycosylation is one of the major post-translational modifications in eukaryote including fungi and mice. Glycosylation can be occurred on Asn residue (N-linked glycosylation) or Ser/Thr residues of polypeptide structures (O-linked glycosylation) and has an impact on characteristics of proteins such as half life, localization and structure [16]. In mice, particular glycan structures on pathogenic fungi are recognized by CLRs as PAMPs [4–7, 9]. However, little is known about whether endogenous glycosylation status can be also sensed by CLRs.

In the present study, we searched for endogenous molecules by using Dectin-2-reporter cells [9] and Dectin-2-Ig-fusion proteins, and identified β-glucuronidase as an endogenous protein recognized by Dectin-2 in BMDCs.

## Materials and Methods

### Experimental animals

C57BL/6 mice were obtained from Japan Clea (Tokyo, Japan). All mice were maintained in a filtered-air-laminar-flow enclosure and given standard laboratory food and water ad libitum. All animal experimental protocols were approved by the committee of Ethics on Animal Experiment, Faculty of Medical Sciences, Kyushu University.

### Generation of *Gusb*^–/–^mice

*Gusb*^–/–^mice were established by CRISPR-Cas9 system with C57BL/6 embryo. Off-target analysis was performed using CRISPRdirect software (https://crispr.dbcls.jp) or CRISPRdesign software (http://crispr.mit.edu). A single-guide RNA sequence used for injection was shown as follows; GAGTGCGTGTTGGGTCGCGC. Genotyping was confirmed by the direct sequencing of this target site.

### Antibodies and flowcytometric analysis

Phycoerythrin-conjugated anti-CD11c mAb (HL3) was from BD Bioscience. Anti-Flag mAbs (1E6 and M2) were from Wako and Sigma-Aldrich, respectively. Anti-actin Ab (4970) was from Cell Signaling Technology. HRP-conjugated polyclonal anti-hIg antibody was from Jackson ImmunoResearch. Polyclonal anti-Gusb Ab was from proteintech. Protein A sepharose beads and streptavidin sepharose beads were from GE healthcare. For the flowcytometric analysis, stained cells with indicated mAbs were acquired on flow cytometer. Viable cells were analyzed after gating on cells negative for Propidium iodide (PI) (Sigma-Aldrich). The data were analyzed using FlowJo software version 10.0.7 (Tree Star).

### Plasmids

Full length of Gusb was cloned by PCR using the following primers; 5’-ATGCGAATTCATGTCCCTAAAATGGAGTGCGTGTTG-3’ (sense) and 5’-CGGAAGCAGACCGTTCACGTTCTAACTCGAGAGCT-3' (anti-sense). Cloned cDNA was inserted into pMx-IRES-human CD8 vector. Flag tag was fused at the C-terminus of Gusb. Mutated forms of Gusb that lacked multiple N-glycosylation sites were generated by substituting four asparagine residues (Asn^172^, Asn^416^, Asn^591^, Asn^627^) into glutamine. Primer pairs used for site directed mutagenesis are follows; for Gusb N^172^Q, 5’- GCCATCCAAAACACACTGACCCCTCATACC-3’ (sense) and 5’- TCAGTGTGTTTTGGATGGCAATCGTGATCC-3' (anti-sense); for Gusb N^416^Q, 5’-GTTTTGGCCAAGAGTCACTTCGGCACCACC-3’ (sense) and 5’-CCGAAGTGACTCTTGGCCAAAACTCTGAGG-3' (anti-sense); for Gusb N^591^Q, 5’-ACTTCATGACGCAACAGTCACCGCTGAGAG-3’ (sense) and 5’- AGCGGTGACTGTTGCGTCATGAAGTCGGCG-3' (anti-sense); for Gusb N^627^Q, 5’-TTGCCCAAGAAACCGGAGGTCACGGTTCAG-3’ (sense) and 5’- CTCCGGTTTCTTGGGCAATCCTCCAGTATC-3' (anti-sense).

### Cells

For the development of BMDCs, bone marrow (BM) cells were collected from femurs, pelvises and fibulas and 1 × 10^6^ cells of BM cells were cultured in RPMI 1640 medium with 10% FBS in the presence of GM-CSF. After the culture, cells were harvested on indicated days. 2B4-NFAT-GFP reporter cells expressing murine Dectin-2 or Dectin-2^QPD^ were prepared as described previously [9]. Gusb-overexpressing RAW264.7 cells were established by retrovirus-mediated gene transfer.

### Analysis of Dectin-2-binding proteins

A plasma membrane fraction of BMDCs was isolated by Minute plasma membrane kit (Invent) following the manufacture’s protocol and then was lysed in NP-40 lysis buffer (1% Nonidet P-40, 50 mM Tris-HCl, 150 mM NaCl, 5 mM EDTA, 10 μg/ml aprotinin, 12.5 μg/ml chymostatin, 50 μg/ml leupeptin, 25 μg/ml pepstatin, and 1 μM PMSF). After rotating of the lysates with protein A sepharose beads at 4°C for 1 h, the beads were collected by centrifugation. Collected beads were washed with NP-40 lysis buffer five times and eluted in 40 μl of SDS sample buffer (125 mM Tris-HCl, 25% of glycerol, 2.5% SDS, 2.5% mercaptoethanol, 0.0025% of bromophenol blue) for heat denaturation, or in 500 μl of PBS containing 0.1 M mannose. Eluted samples were separated by SDS-PAGE, and were visualized by Sil-Best Stain one (nacalai tesque). Samples were analyzed by Mass spectrometry Orbitrap Velos Pro (Thermo Fisher Scientific). Obtained data was analyzed by using Mascot search engine (http://www.matrixscience.com).

### Generation of Fc-fusion proteins

Extracellular domains of Dectin-2 or Dectin-2^QPD^ (a.a. 43–209) were fused to N-terminus of human IgG1 Fc region into pDisplay vector (invitrogen). pDisplay-Ig, pDisplay-Dectin-2-Ig, pDisplay-Dectin-2^QPD^-Ig were transfected into HEK293T cells by Polyethylenimine Max (Polyscience). 10 days after transfection, supernatants were collected and purified by Profinia (Bio-Rad). 500 μg of Fc-fusion proteins was used for affinity precipitation.

## Results

### Myeloid cells express molecule(s) recognized by Dectin-2

**Fig 1:**
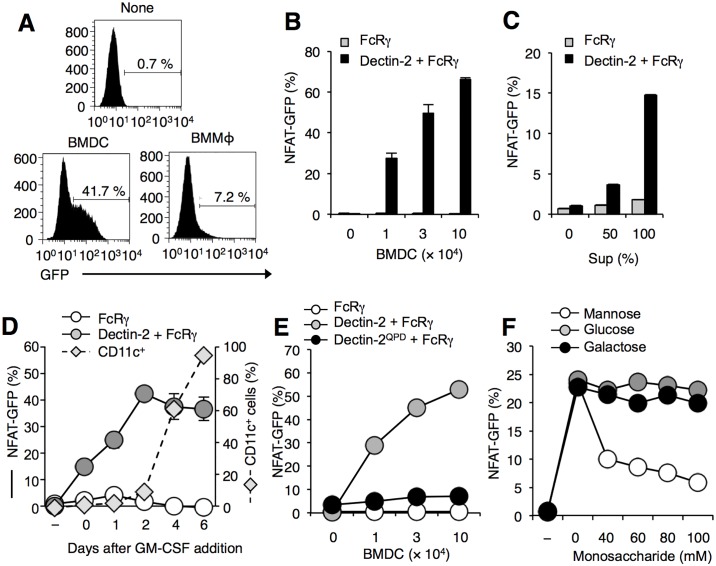
Dectin-2 recognizes endogenous molecule(s) on BMDCs. (A-D) Dectin-2 recognized molecules derived from BMDCs. (A) Reporter cells expressing Dectin-2 together with FcRγ were incubated with or without BMDCs or BMDMs for 18 h. Numbers in histograms showed the percentage of NFAT-GFP + cells after the culture. (B, C) Reporter cells expressing FcRγ alone (grey square), FcRγ together with Dectin-2 (black square) were incubated (B) with indicated cell numbers or (C) with culture supernatants of BMDCs for 18 h. (D) Changes of CD11c^+^ cells (dot line) and NFAT-GFP + cells (solid line) during the differentiation of BMDCs were shown. (E and F) Essential role of mannose-binding motif of Dectin-2 for recognition of molecules in BMDCs. (E) Reporter cells expressing FcRγ alone (open circle), FcRγ together with Dectin-2 (grey circle) and FcRγ together with Dectin-2^QPD^ (black circle) were incubated with indicated cell numbers of BMDCs for 18 h. (F) Reporter cells expressing Dectin-2 together with FcRγ were incubated with 2 × 10^5^ of BMDCs in the presence of indicated concentrations of mannose (open circle), glucose (grey circle) or galactose (black circle) for 18 h. These data are presented as the means ± S.D. for duplicate assays, and representative of at least three independent experiments.

To search for molecules recognized by CLRs, Dectin-2-expressing reporter cells were cultured with BMDCs or bone marrow-derived macrophages (BMDMs) and then GFP expression was analyzed as a read-out [9]. As a negative control, FcRγ-expressing reporter cells were used. As shown in Fig 1A, GFP expressions on Dectin-2-reporter cells were induced after the co-culture with both BMDCs and BMDMs. BMDCs had a stronger activity to induce the GFP expression than those in BMDMs (Fig 1A). The GFP expression levels on Dectin-2-reporter cells correlated with the number of BMDCs (Fig 1B). Culture supernatants of BMDCs also had an activity to induce the GFP expression, implying that Dectin-2 might recognize molecules released into the culture medium (Fig 1C). We next asked whether molecules recognized by Dectin-2 were induced during differentiation of BMDCs. Before the differentiation of BMDCs, bone marrow cells had relatively weak activity to induce GFP expression on Dectin-2 reporter cells. After addition of GM-CSF, the GFP expression was enhanced as the percentage of CD11c^+^ DCs increased (Fig 1D). These results suggest that Dectin-2 recognizes molecules which are mainly expressed on BMDCs.

Since CLRs are defined as carbohydrates-binding proteins, we next asked whether carbohydrates were involved in Dectin-2 recognition of molecules in BMDCs. EPN motif, which is mannose-binding motif for C-type lectin, is crucial for Dectin-2 recognition to α-mannan [9, 17]. To test whether the motif was involved in recognition of molecules on BMDCs, we set out two experiments. First, we generated reporter cells harboring mutated EPN motif to galactose-binding motif in Dectin-2 (Dectin-2^QPD^). After the co-culture of Dectin-2^QPD^ with BMDCs, GFP expression was not induced and the expression level was comparable to that of FcRγ-expressing control cells (Fig 1E). Second, to examine whether blocking of mannose binding in EPN motif had an impact on interaction between Dectin-2 and the BMDCs, an excessive amount of mannose was added during the co-culture. We found that blocking of EPN motif by addition of mannose, but not other monosaccharides such as glucose and galactose, greatly reduced Dectin-2-mediated ligand recognition activity as judged by GFP expressions (Fig 1F). These results suggest that Dectin-2 may recognize BMDCs through mannose-related structure.

### β-glucuronidase is an endogenous molecule bound to Dectin-2

**Fig 2:**
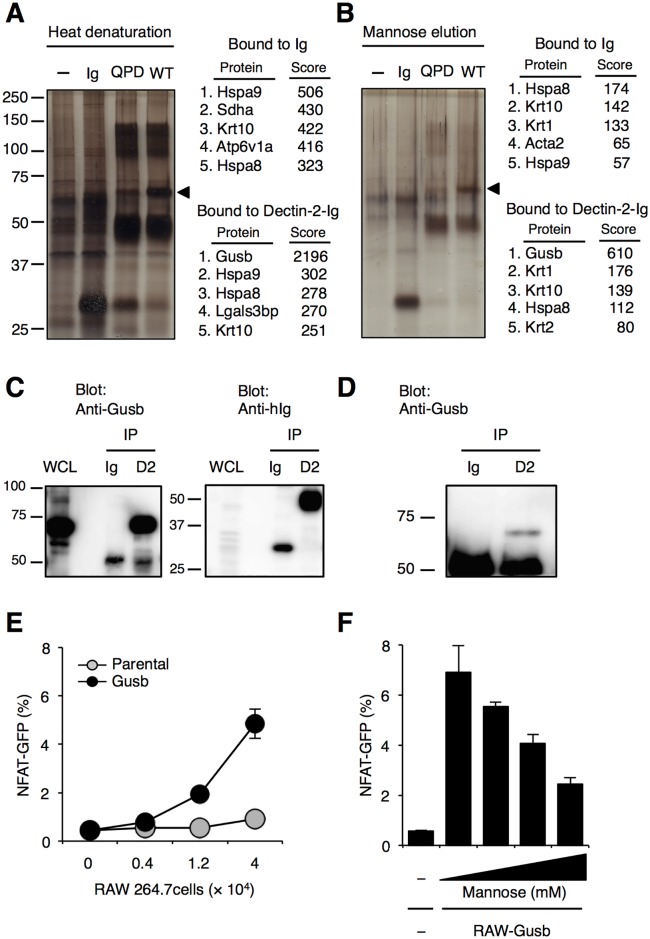
Dectin-2 binds to Gusb. (A and B) Identification of Dectin-2-binding molecules on BMDCs. Affinity-purified proteins using Ig, Dectin-2^QPD^-Ig (QPD) and Dectin-2-Ig (WT) were eluted with (A) heating or (B) addition of elution buffer containing 100mM Mannose. Top 5 proteins with high Mascot score were listed on the right side of the pictures. (C, D) Gusb expressions in BMDCs. Affinity-precipitated fractions with Ig and Dectin-2-Ig (D2) from whole cell lysate (WCL) of BMDCs (C) or culture supernatants (D) were analyzed by western blotting using anti-Gusb or anti-hIg mAb. (E) Reporter cells expressing FcRγ together with Dectin-2 were incubated with indicated cell numbers of Gusb-overexpressing RAW264.7 cells (black circle) or parental cells (Grey circle). (F) Dectin-2 reporter cells were incubated with Gusb-overexpressing RAW264.7 cells in the presence of mannose (100, 80, 60, 40 mM from the right) for 18 h. These data are presented as the means ± S.D. for duplicate assays, and representative of at least two independent experiments.

To search for the candidate molecules recognized by Dectin-2, we generated a soluble protein harboring extracellular domain of Dectin-2 or Dectin-2^QPD^, which was fused to the carboxyl terminus of the human immunoglobulin Fc region (Dectin-2-Ig or Dectin-2^QPD^-Ig). By using Dectin-2-Ig as a probe, Dectin-2-binding molecules were purified from lysates of BMDCs. The candidate proteins specifically bound to Dectin-2-Ig, but not beads itself, Ig only and Dectin-2^QPD^-Ig, were detected around 75 kilo-Dalton (kD) (Fig 2A). We observed similar size of the bands when Dectin-2-binding proteins were eluted by addition of an excessive amount of mannose (Fig 2B). The band at 75 kD was excised and analyzed by Mass spectrometry. Further analysis of the band with Mascot search engine (http://www.matrixscience.com) revealed that this fraction contained β-glucuronidase (Gusb), Hspa9, Hspa8, Lgals3bp, Krt10 and, based on the Mascot score, Gusb had the highest probability among the detected proteins (Fig 2A and 2B). Hspa9, Hspa8, Lgals3bp and Krt10 were also detected in the band at 75kD from samples purified with Ig. These results suggest that Gusb was the most probable candidate protein bound to Dectin-2. Indeed, Gusb is highly expressed in BMDCs, and was precipitated by Dectin-2-Ig, but not Ig (Fig 2C). Consistent with the observation that culture supernatants of BMDCs activated Dectin-2-expressing cells (Fig 1C), Gusb was also detected in the supernatants by Dectin-2-Ig (Fig 2D). In order to examine whether Gusb expression was sufficient for being recognized by Dectin-2, Gusb was transduced into macrophage cell line RAW264.7 cells and co-cultured with Dectin-2-expressing reporter cells. GFP expression in Dectin-2-expressing reporter cells was significantly enhanced after the co-culture with Gusb-overexpressing RAW264.7 cells as compared with parental cells (Fig 2E) and this activity was reduced in the presence of an excess amount of mannose (Fig 2F). These results indicate that Gusb is an endogenous protein recognized by Dectin-2.

### Glycosylation on β-glucuronidase is involved in binding of Dectin-2

**Fig 3:**
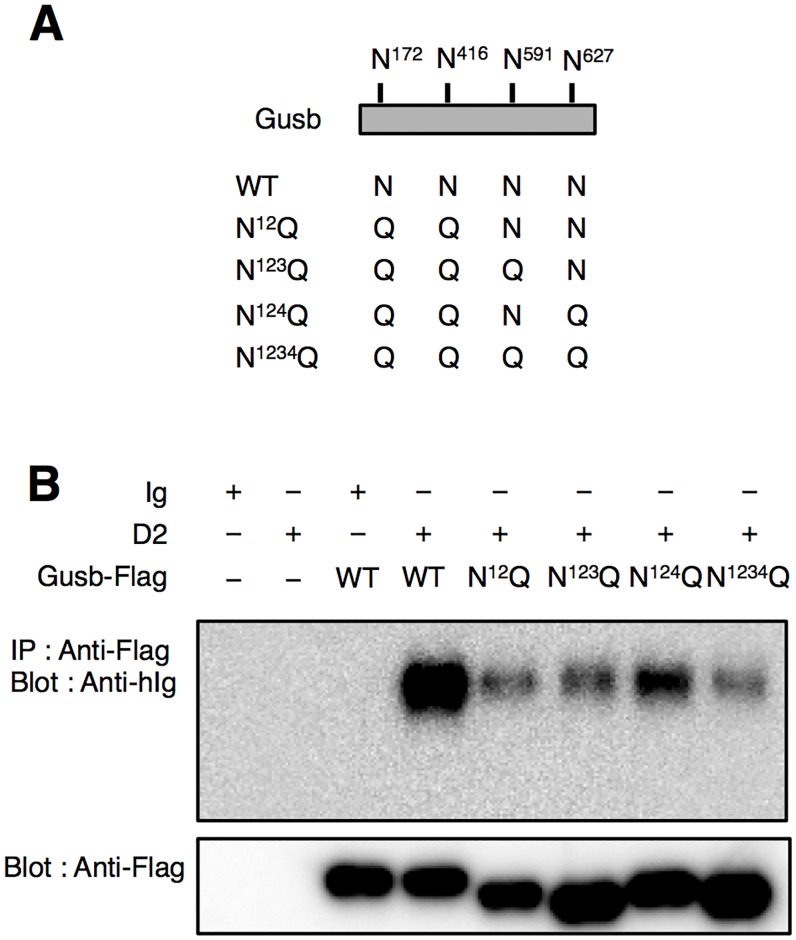
Glycosylation on Gusb is important for binding of Dectin-2. (A) Schematic diagram of N-glycosylation sites on Gusb. (B) The importance of glycosylation for binding of Dectin-2. Flag-tagged Gusb were immunoprecipitated with biotinylated anti-Flag (M2) antibody followed by enrichment with streptavidin beads. After incubation of Flag-tagged Gusb proteins with Dectin-2-Ig, co-precipitated Dectin-2-Ig was detected by western blotting using anti-hIg mAb.

It is previously reported that Gusb possesses four consensus sequences for N-linked glycosylation (a.a. 172–174, Asn-Asn-Thr; 416–418, Asn-Glu-Ser; 591–593, Asn-Gln-Ser; 627–629, Asn-Glu-Thr) and N-glycosylated Gusb has high mannose type N-glycan [18, 19]. To examine the importance of glycosylation of Gusb in Dectin-2 recognition, a series of Flag-tagged Gusb with mutated N-linked glycosylation sites was generated (Fig 3A). Dectin-2 binding activity of Gusb was reduced if the glycosylation sites were mutated (Fig 3B), suggesting the involvement of glycosylation in Dectin-2 recognition.

### Dectin-2 binds several endogenous molecules including Gusb

**Fig 4:**
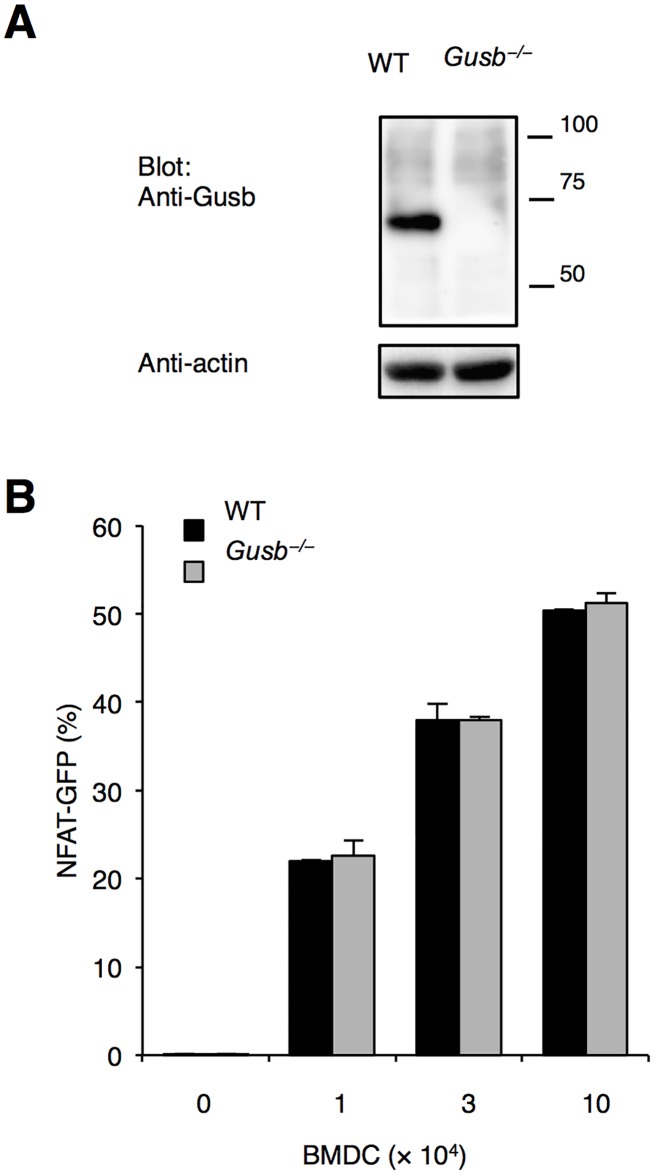
Generation of Gusb-deficient mice. (A) *Gusb*^–/–^mice were established by CRISPR-Cas9 system. Expression of Gusb on BMDCs was analyzed by western blotting. (B) Dectin-2-reporter cells recognized Gusb-deficient BMDCs. Reporter cells expressing Dectin-2 together with FcRγ were incubated with WT (black bar) or Gusb-deficient (grey bar) BMDCs for 18 h.

Finally, to test the contribution of Gusb as endogenous ligands for Dectin-2, we generated *Gusb*^–/–^mice by using CRISPR-Cas9 system (Fig 4A). *Gusb*^–/–^mice were smaller than wild-type littermates as previously reported [20, 21]. We found that Dectin-2-reporter cells were activated when co-cultured with Gusb-deficient BMDCs, suggesting that Gusb is not a sole protein and other molecules can be sensed by Dectin-2 (Fig 4B).

## Discussion

In the present study, we identified an endogenous protein Gusb as a Dectin-2-binding protein. However, the specificity of protein ligands for Dectin-2 is still an open question. As Gusb-deficient BMDCs were capable of activating Dectin-2-reporter cells, other protein(s) are likely to provide the same particular glycans to be recognized by Dectin-2. Given that Gusb is one of the most abundant proteins in myeloid cells [22] but not lymphocytes (data not shown), more detail analysis using sensitive Mass spectrometry may identify other “career” proteins which are less abundantly expressed. Although the precise glycan structure on Gusb has not been determined yet, our data suggest that mannose is likely to be involved in the recognition by Dectin-2 (Fig 1F).

It has been reported that Dectin-2 recognizes terminal mannose of N-linked glycans in fungi [5, 7]. However, recent studies showed that Dectin-2 also recognizes lipomannan, mannoprotein and mannosylated O-antigen that contain terminal mannose [9, 11, 23]. Interestingly, in vertebrates, high mannose-type N-linked glycans are also found on particular type of DC [24]. Gusb is known to contain high mannose type glycans [18]. These observations suggest that, irrespective of pathogens and host tissues, mannose moieties which are found in proteins, glycans, O-antigens or any kinds of scaffold, could be recognized by Dectin-2.

Glycosylation is one of major post-translational modifications [16, 25]. Our results showed that Dectin-2 binding of Gusb was not completely abrogated if all of N-glycosylation sites were mutated. We found that Gusb has putative O-glycosylation sites which might be recognized by Dectin-2 as previously described (http://www.cbs.dtu.dk/services/NetOGlyc/) [9]. This observation suggests that Dectin-2 may bind to O-linked glycan on Gusb. We also could not exclude the possibility that Gusb protein itself is involved in Dectin-2 binding.

Accumulating evidence has revealed that Dectin-2 contributes to host defense against pathogens. In addition to protective effects against pathogens, it is proposed that Dectin-2 plays a role in protecting the host by dampening the excessive inflammatory responses. It was reported that putative Dectin-2 ligand was expressed on regulatory T cells and blockage of Dectin-2-mediated signaling broke the immune tolerance [26]. We previously reported that Dectin-2 is essential for production of anti-inflammatory cytokine, IL-10 in BMDCs [11]. *Gusb*^–/–^mice spontaneously develop Mucopolysaccharidoses (MPS) accompanied by overexpression of inflammatory cytokines [27–29]. It is interesting to see whether the lack of such Dectin-2-mediated inhibitory effects is involved in the development of the disease in *Gusb*^–/–^mice.

Some N-glycan on Gusb contains a terminal mannose 6-phosphate (M6P), which is important for lysosomal enzymes to localize in lysosome. Gusb is also known to be released into culture supernatants [19, 30] and we also confirmed this in BMDCs (Fig 2D). Although the exact location in which Gusb is sensed by Dectin-2 are yet uncharacterized, Dectin-2 might sense Gusb both on cell surface and in the extracellular milieu. The possibility that Gusb reflects cellular damages would be an intriguing issue to be addressed in the future.

Recently, it is widely accepted the idea of immune surveillance—normal and abnormal self are continuously monitored by our immune systems. In line with this concept, Dectin-2 may monitor abnormal glycosylation status of several proteins exposed on the cell surface. This “status” may include the exposure of terminal mannose due to aberrant loss of sialic acid or high-mannose structure which should be only present in intracellular organelles. Interestingly, fungus and yeast normally express high-mannose glycan on the cell surface. Thus, it is tempting to speculate that Dectin-2, and presumably other CLRs, may sense “danger” derived from self and non-self through similar criteria, which warrants further extensive investigation.
